# Supplementary material for: Listeria monocytogenes cell-to-cell spread in epithelia is heterogeneous and dominated by rare pioneer bacteria
Source: eLife. 2019 Feb 5;8:e40032. doi: 10.7554/eLife.40032 (PMC6363384; doi:10.7554/eLife.40032)
Supplement: Transparent reporting form [file elife-40032-transrepform.docx]

***eLife’s* transparent reporting form**

We encourage authors to provide detailed information *within their submission* to facilitate the interpretation and replication of experiments. Authors can upload supporting documentation to indicate the use of appropriate reporting guidelines for health-related research (see [EQUATOR Network](http://www.equator-network.org/%20)), life science research (see the [BioSharing Information Resource](https://biosharing.org/)), or the [ARRIVE guidelines](http://www.plosbiology.org/article/info:doi/10.1371/journal.pbio.1000412) for reporting work involving animal research. Where applicable, authors should refer to any relevant reporting standards documents in this form.

If you have any questions, please consult our Journal Policies and/or contact us: [editorial@elifesciences.org](mailto:editorial@elifesciences.org).

**Sample-size estimation**

- You should state whether an appropriate sample size was computed when the study was being designed
- You should state the statistical method of sample size computation and any required assumptions
- If no explicit power analysis was used, you should describe how you decided what sample (replicate) size (number) to use

Please outline where this information can be found within the submission (e.g., sections or figure legends), or explain why this information doesn’t apply to your submission:

All data points for experimental data were plotted. For simulated data, around 50 data points were shown out of 1000 that were created (for simplicity). This information can be found in the figure legends.

All p-values were calculated with non-parametric tests (Wilcoxon rank sum test for comparing two groups to each other or Kruskal-Wallis test for comparing multiple groups). This information can be found in the figure legends.

The statistical tests used in this study were non-parametric tests that compared the mean. As many replicates as possible were collected (>30 data points per experiment) before running the tests.

**Replicates**

- You should report how often each experiment was performed
- You should include a definition of biological versus technical replication
- The data obtained should be provided and sufficient information should be provided to indicate the number of independent biological and/or technical replicates
- If you encountered any outliers, you should describe how these were handled
- Criteria for exclusion/inclusion of data should be clearly stated
- High-throughput sequence data should be uploaded before submission, with a private link for reviewers provided (these are available from both GEO and ArrayExpress)

Please outline where this information can be found within the submission (e.g., sections or figure legends), or explain why this information doesn’t apply to your submission:

Extensive details on how data were collected and analyzed are provided in Materials and Methods.

The difference between “biological” and “technical” replicates does not apply in this study as each infection focus is independently derived from a single invasion event, and each simulation is independent of every other. We describe in the figure legends that data collected on different experimental days were plotted with different shapes (i.e. circles, squares, triangles). We clearly explain in the figure legends what each data point represents (i.e. a focus, or the average of n number of foci, etc).

All data, with exception of randomly selected simulated data (see answers in sample-size estimation) were plotted.

**Statistical reporting**

- Statistical analysis methods should be described and justified
- Raw data should be presented in figures whenever informative to do so (typically when N per group is less than 10)
- For each experiment, you should identify the statistical tests used, exact values of N, definitions of center, methods of multiple test correction, and dispersion and precision measures (e.g., mean, median, SD, SEM, confidence intervals; and, for the major substantive results, a measure of effect size (e.g., Pearson's r, Cohen's d)
- Report exact p-values wherever possible alongside the summary statistics and 95% confidence intervals. These should be reported for all key questions and not only when the p-value is less than 0.05.

Please outline where this information can be found within the submission (e.g., sections or figure legends), or explain why this information doesn’t apply to your submission:

Statistical analysis methods are defined in the figure legends.

All raw data are shown as individual data points (with few exceptions, clearly described in the figure legends). Whenever N <= 100, all data points were plotted.

The two methods we used are the well-defined non-parametric Wilcoxon rank sum test and the Kruskal-Wallis test. Each test was applied to the data shown in the figures.

All significant p-values are shown in the figures. When p > 0.05, ‘ns’ was used.

(For large datasets, or papers with a very large number of statistical tests, you may upload a single table file with tests, Ns, etc., with reference to sections in the manuscript.)

**Group allocation**

- Indicate how samples were allocated into experimental groups (in the case of clinical studies, please specify allocation to treatment method); if randomization was used, please also state if restricted randomization was applied
- Indicate if masking was used during group allocation, data collection and/or data analysis

Please outline where this information can be found within the submission (e.g., sections or figure legends), or explain why this information doesn’t apply to your submission:

Microscopy data were randomly collected using µManager’s High Content Screening (HTC) site generator.

Simulated data were generated computationally. When 1000 simulations were run, 50 points at random were selected to depict in the figure (for clarity), but the average of the 1000 simulations was always comparable to the random sample of the 50 data points shown.

**Additional data files (“source data”)**

- We encourage you to upload relevant additional data files, such as numerical data that are represented as a graph in a figure, or as a summary table
- Where provided, these should be in the most useful format, and they can be uploaded as “Source data” files linked to a main figure or table
- Include model definition files including the full list of parameters used
- Include code used for data analysis (e.g., R, MatLab)
- Avoid stating that data files are “available upon request”

Please indicate the figures or tables for which source data files have been provided:

We uploaded code used to produce simulated data (in MATLAB) to a GitHub account at https://github.com/Fabianjr90.
